# EMGD-FE: an open source graphical user interface for estimating isometric muscle forces in the lower limb using an EMG-driven model

**DOI:** 10.1186/1475-925X-13-37

**Published:** 2014-04-04

**Authors:** Luciano Luporini Menegaldo, Liliam Fernandes de Oliveira, Kin K Minato

**Affiliations:** 1 Alberto Luiz Coimbra Institute for Graduate Studies and Research in Engineering, Biomedical Engineering Program (PEB/COPPE), Federal University of Rio de Janeiro, Av. Horácio Macedo 2030, Bloco H-338, 21941-914 Rio de Janeiro, RJ, Brazil; 2 School of Physical Education and Sports, Federal University of Rio de Janeiro, Av. Carlos Chagas Filho 540, 21949-900 Rio de Janeiro, RJ, Brazil; 3 PETROBRAS S/A, Rio de Janeiro, Brazil

**Keywords:** Muscle biomechanics, EMG-driven models, Muscle force estimation, Lower limb muscles

## Abstract

**Background:**

This paper describes the “EMG Driven Force Estimator (EMGD-FE)”, a Matlab® graphical user interface (GUI) application that estimates skeletal muscle forces from electromyography (EMG) signals. Muscle forces are obtained by numerically integrating a system of ordinary differential equations (ODEs) that simulates Hill-type muscle dynamics and that utilises EMG signals as input. In the current version, the GUI can estimate the forces of lower limb muscles executing isometric contractions. Muscles from other parts of the body can be tested as well, although no default values for model parameters are provided. To achieve accurate evaluations, EMG collection is performed simultaneously with torque measurement from a dynamometer. The computer application guides the user, step-by-step, to pre-process the raw EMG signals, create inputs for the muscle model, numerically integrate the ODEs and analyse the results.

**Results:**

An example of the application’s functions is presented using the *quadriceps femoris* muscle. Individual muscle force estimations for the four components as well the knee isometric torque are shown.

**Conclusions:**

The proposed GUI can estimate individual muscle forces from EMG signals of skeletal muscles. The estimation accuracy depends on several factors, including signal collection and modelling hypothesis issues.

## Background

Estimating skeletal muscle forces *in vivo* is a challenging problem in biomechanics. The possibilities for directly measuring such forces are limited due to the invasive nature of the procedure. Inverse multibody dynamics, associated with static or dynamic optimisation, can produce an estimate of a muscle’s dynamic state [1]; however, the results are strongly dependent on the solution of the muscle force-sharing problem [2]. EMG-driven modelling is an attractive technique to estimate muscle forces *in vivo* for both healthy and pathological conditions [3]–[12]. The basic idea consists of using electromyography (EMG) signals as the inputs of a Hill-type muscle model. A specific characteristic of the EMG-driven model used in EMGD-FE relies on that it is formulated entirely in terms of ordinary differential equations (ODEs) to represent muscle dynamics [6]. Numerical integration of the ODEs yields the dynamic state of the muscle model. In our case, it comprises the muscle active state (activation), the tendon force and the length of the contractile part of the musculotendon unit.

Compared with force sharing solutions obtained through optimisation, EMG-driven models do not require the definition of an *a priori* cost function and they have a low numerical cost and converge easily, especially when compared with dynamic optimisation [1]. In addition, EMG-driven models take into account muscle dynamics, which is usually neglected in static optimisation [13]. However, EMG techniques present some well-known limitations that apply to EMG-driven models as well. Deep muscles are not accessible by surface electrodes, and large-scale problems require numerous EMG channels. The relationship between the collected bipolar EMG and the real degree of muscle activity is not trivial, and it can be subjected to a number of misinterpretations [14]. Considering such limitations, bipolar EMG is still a very useful tool to assess the degree of muscle activity. In the best case, a rectified, MVC-normalised and filtered EMG gives a reasonable estimation of muscle excitation timing and amplitude, but it provides no force information. Considering muscle mechanics models further extend the utility of an EMG analysis, allowing the estimation of muscle force.

Comprehensive predictive simulations—such as the solution of a dynamic optimisation/optimal control problem—of muscle activity during a motor task cannot be performed by EMG-driven models; however, forces estimated using experimental EMG data can be used to constrain the solutions of the optimisation problem to a more physiologically feasible space.

This paper presents a graphical user interface (GUI) that estimates muscle forces using a dynamic Hill-type EMG-driven model (EMG Driven Force Estimator (EMGD-FE) v1.0). It is implemented as an open source Matlab® graphical user interface (GUI) under general public license (GPL). Essentially, it automates the process that has been used by our group to process EMGs and to pass them into a dynamic model of skeletal muscles, which is then numerically integrated. The resulting muscle forces are multiplied by their respective moment arms, and the partial joint torques are totalled and compared to a joint torque curve, obtained by inverse dynamic analysis or dynamometry. Finally, the results are plotted and analysed^a^. All the processing steps are performed through sequential and self-explanatory GUI windows, which allow the next step to be performed only when the previous one is accomplished, thereby reducing mistakes. A set of default parameters for the lower limb is supplied and can be easily edited in a set of Extensible Markup Language (XML) files. Up to 6 Generic Muscles, without pre-defined parameters, can be used to analyse unlisted muscles.

Several muscle-mechanics formulations can be used for EMG-driven studies. Here, we follow the analytical and the numerical procedures used in our own muscle biomechanics research, which include model formulation, parameter estimation, data collection and filtering characteristics. In the current GUI implementation, no parameter optimisation algorithm for the adjustment of torque curves is available.

An example involving *quadriceps femoris* is included for illustrative purposes. Our intention is to provide periodic updates to the GUI that incorporate new features and improvements into the muscle dynamics model, the EMG collection steps and the processing procedures. One major limitation of the current version is that only isometric analyses can be performed. For non-isometric muscle operation, the model is particularly sensitive to certain parameters, such as the tendon slack length, the maximum force and the pennation angle. From preliminary simulations [15], we observed that in vivo calibration using both numerical optimisation as well as more sophisticated muscle and tendon material constitutive equations are necessary to obtain reliable force estimates. This feature will be included in future versions of the GUI.

## Implementation

### Muscle dynamics, data collection and EMG to force processing

The Hill-type dynamic muscle model comprises contractile, parallel elastic and parallel damping elements, as well as an elastic tendon. It is a modified version of the Zajac [16] model, incorporating parallel elastic and damping elements with the main objective of improving numerical stability. It has three dynamic states, activation (*a*), tendon force (*F*^*T*^) and muscle length (*L*^*M*^), and two inputs excitation (*u*) and musculotendon velocity (*V*^*MT*^). The last input variable was set to zero because the contractions are isometric. The ODEs (Eq. 1) are integrated using a variable step-size Runge–Kutta algorithm. The first line of Eq. 1 represents the activation dynamics, and the second represents the contraction dynamics. The last line allows the integration of the muscle velocity to explicitly produce the muscle length. This state variable is used in a polynomial function that is part of the contraction dynamics, which relates the muscle maximal force and length. The model has been used extensively in a number of published studies, and its analytical derivation is found in Menegaldo and Oliveira [7].

(1) $$f\left(x,u\right)=\left[\begin{array}{c} \dot{a}\\ {\dot{F}}^{T}\\ {\dot{L}}^{M} \end{array}\right]=\left[\begin{array}{c} \left(u-a\right)\left(k_{1}u+k_{2}\right)\\ g\left(F^{T},a,u,L^{M}\right)\\ V^{M}=\frac{dL^{M}}{\mathrm{dt}} \end{array}\right]$$

**Figure 1:**
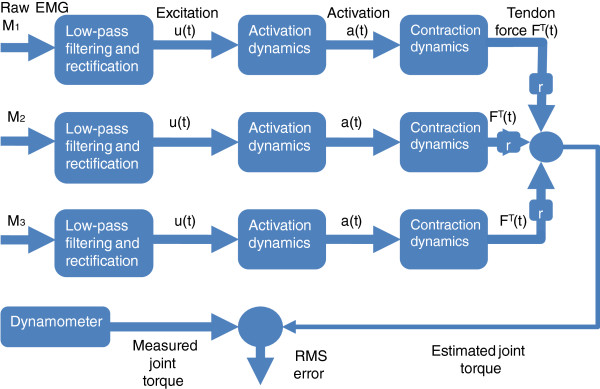
**EMG-driven model for muscle force estimation.** The EMG for muscles M_1,2,3_ (or more) is rectified and low-pass filtered for envelope extraction. Neuromuscular excitation, u(t), is the input for the activation dynamics. Activation, a(t), drives the contraction dynamics, which is integrated to produce the tendon force. The joint torque, which is compared to the joint torque measured by the dynamometer, is estimated by the sum of the tendon forces multiplied by their respective moment arms (either positive or negative, depending on if the muscle actuates as an extensor or a flexor).

The EMG-driven force estimation procedure comprises the following: collecting EMG signals, followed by rectifying, filtering and numerically integrating the muscle model based on this input. The resulting tendon force is multiplied by the muscle moment arm, and the sum of the individual muscles’ contributions to the total torque can be compared to the simultaneously collected dynamometer measurements (Figure 1). Moment arm values depend on the joint angle that the isometric experiment was performed, and should be provided by the user. They can be easily estimated using Opensim [17] or polynomial regression equations [18].

**Figure 2:**
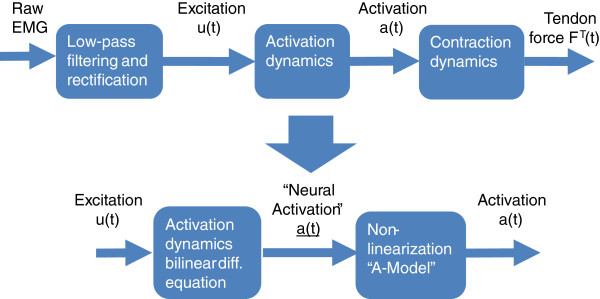
**Activation dynamics.** Its implementation comprises two steps. The first is the second row of eq. (1), and the second is a non-linear algebraic equation, which augments low-level activations and is modulated by a single “A” parameter [19].

In the current implementation, the activation dynamics comprises two steps: a differential bilinear equation and an algebraic non-linearisation operation (“A-model”, Figure 2), which is modulated by a single “A” parameter [19]. This model slightly augments low-level activations relative to high-level ones, representing relationships between experimentally observed EMG and isometric forces [20]. The effect on the prediction accuracy, by changing the “A” parameter in the EMG-driven model, for the quadriceps muscle has been addressed in Menegaldo and Oliveira [21,22].

The muscle contraction protocol, such as ramp, step, or sine, is freely chosen by the user provided that the contractions are isometric. Electrodes can be either bipolar or multi-channel, but a single raw EMG signal should be provided for each muscle. The maximum voluntary contraction (MVC) torque and EMG, as well as the data of the submaximal tests, should be collected without changing the electrodes or the subject’s position.

### Procedure of analysis using the EMGD-FE

**Figure 3:**
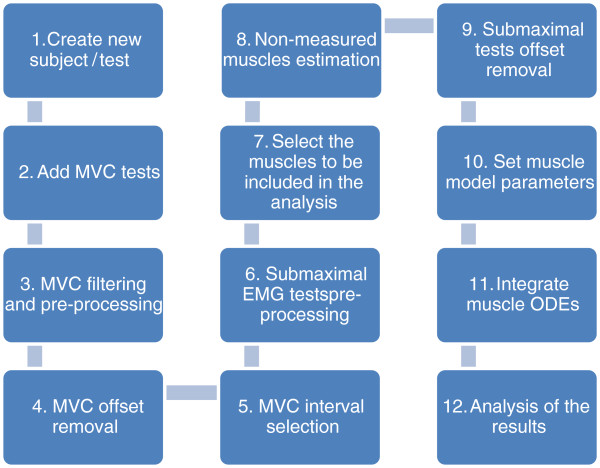
Procedure implemented in the EMGD-FE GUI for estimating the forces based on EMG signals.

**Figure 4:**
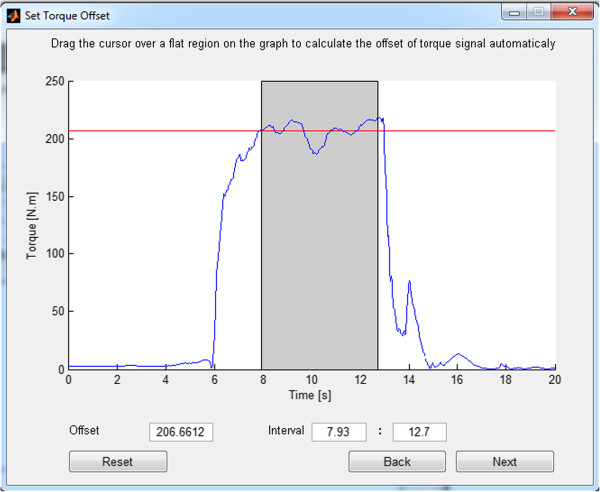
Selection of the maximum torque region to normalise EMG inputs by MVC EMG.

**Figure 5:**
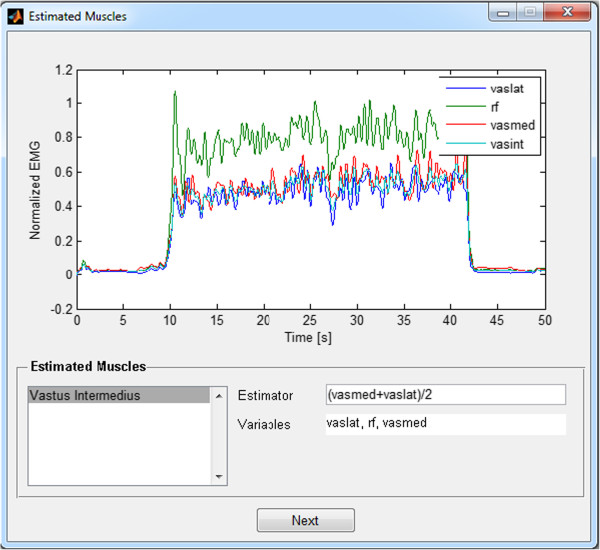
Estimation of the unmeasured EMG signals of deep muscles through the application of a user-defined formula that may depend on the EMG signals from other muscles.

**Figure 6:**
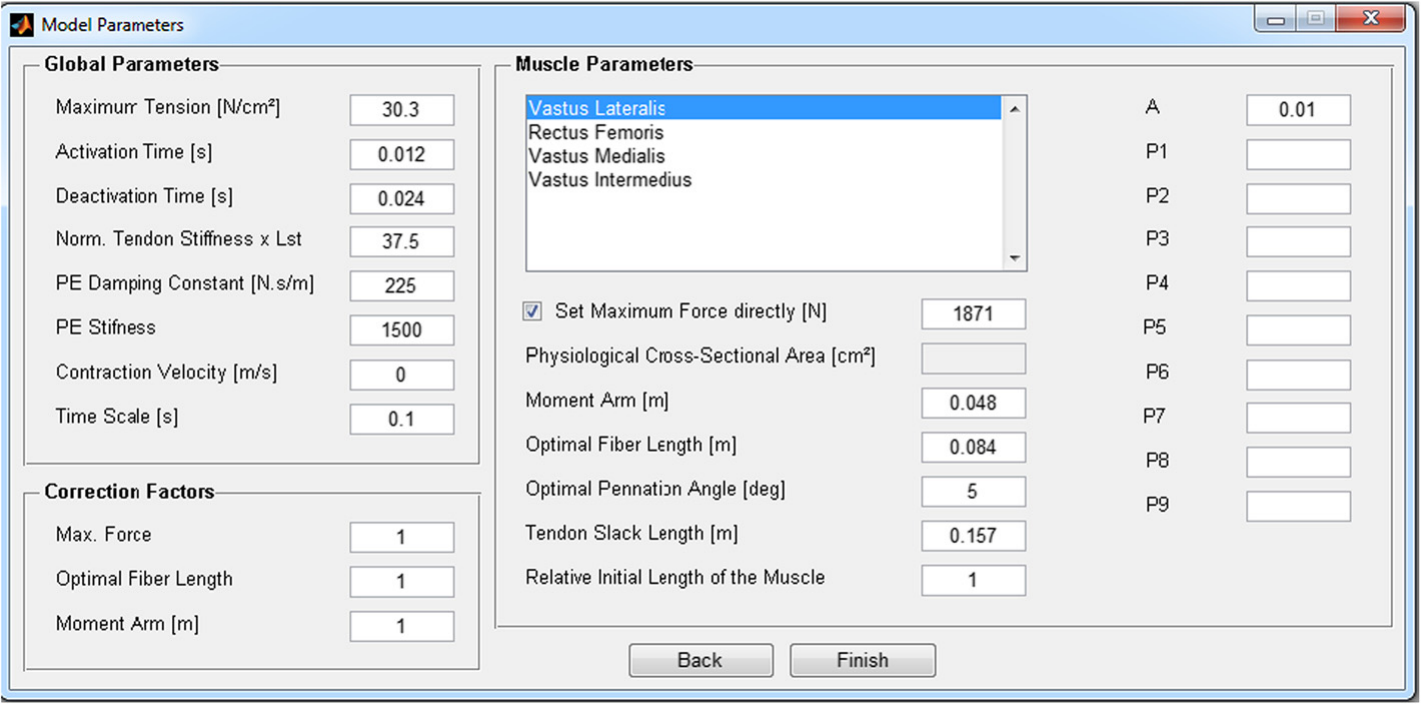
**Muscle model parameter editing window.** Default parameters defined in an XML file are uploaded automatically and can be changed by the user. In the right-side panel, generic parameters are defined and made available to the ‘model.m’ file. This function is integrated using the Runge–Kutta algorithm.

The procedure for using EMGD-FE is summarised in Figure 3 and is described below. The user is guided to provide the necessary information and to choose the appropriate analysis parameters for each case, such as the digital filter design, the selection of the signal time window that will be considered, and the muscle model parameters. Screenshots from selected windows are shown to illustrate the application’s interface (Figures 4, 5, and 6). More details can be found in the EMGD-FE user’s manual.

1. Provide basic information about the subject and the collected data. A *struct* variable “.emg” is created;

2. A text file containing the MVC EMG and torque data must be provided. The data can be organised into a single or separate file(s). The MVC signal is expected to contain an initial timespan of a relaxed EMG (for a few seconds), an intermediate range with the maximum voluntary activation (approximately 5 seconds to avoid fatigue) and an additional relaxing timespan. Within this range, the torque signal has to be sustained for about 2 seconds. Curve shape with a sharp EMG peak should be avoided. These intervals are useful for setting the initial conditions of the ODEs’ states and checking for the quality of the EMG baseline;

3. Rectify and apply zero-lag (forward and backward) filters to the MVC EMG signal: band-pass to remove artefacts, mains hums filter^b^ (50 or 60 Hz) and its three first harmonics) and a low pass filter to extract the signal envelope. The joint torque signal, measured through a dynamometer synchronised with the EMG, is also smoothed by the digital filtering. EMG and dynamometer sampling frequency should be compatible with EMG hardware low-pass filter, i.e. greater than twice analog filter cut-off frequency. Several digital filter parameters can be setup by the user;

4. The user visually selects a signal interval in which activation is approximately zero. Its average EMG level is subtracted from the entire signal, providing a DC-level compensation;

5. The user selects another MVC reference signal region such that the average EMG level of this region is used to normalise the subsequent sub-maximal EMG inputs.. This epoch should last approximately 2 seconds, such that the torque value is stable in the vicinity of its maximum plateau [23], as shown in Figure 4;

6. The MVC procedure is repeated for the sub-maximal tests. The rectified and filtered sub-maximal EMG signals are normalised by the processed MVC EMG, as described above;

7. Select the muscles, which cross the same joint, to be included in the analysis;

8. Estimate deep muscles with unmeasured EMGs by providing a user-defined formula that may depend on the EMG signals from other muscles (Figure 5);

9. Remove the offset. The user selects a signal range with zero activation, which will be subtracted from the whole signal. The user must be sure to record a portion of the EMG (approximately 5 seconds) with the muscles relaxed;

10. Set muscle model parameters. Default values, using data from the Opensim lower limb model [17], are loaded from a XML file. A window shows the nominal parameters for each muscle that the user is allowed to change (Figure 6). Scaling factors can be set for certain parameters (left lower part of the window). In the right-side panel, a list of arbitrary parameters (P1-P9) can be defined, which will be passed to the Matlab m-function where the muscle model is defined. It allows some flexibility for the user changing the model equations without altering the GUI structure. In the current distribution, the parameters P1, P2 and P3 are used to set fixed or variable pennation angle relationships with joint angle, only for *triceps surae* muscle (see EMG-FE User Guide, Sec, 2.6.1. Setting Muscle Parameters, for details). The other parameters (P4-P9) have no use in the current version. Additionally, the activation dynamics “A” parameter is defined;

11. Integration of the muscle dynamics ODEs. The model is written in a Matlab m-function called by the “ODE-45” Runge–Kutta integrator. The function where the model is defined has simple syntax, and the user can easily define or modify his/her model formulation. The memory requirements and the simulation time are small based on the fact that the simulations run in only a few seconds on an ordinary laptop. Alternative muscle dynamics formulations that lead to stiff equations can take advantage of an appropriate ODE integration algorithm. Matlab provides a number of alternative integrators, which can easily replace the ODE45 using the same syntax.

12. Several output options are available: the raw and processed EMG signals, the muscle forces, the muscle contributions to total torque, and the torque reproduction error.

## Results and discussion

**Figure 7:**
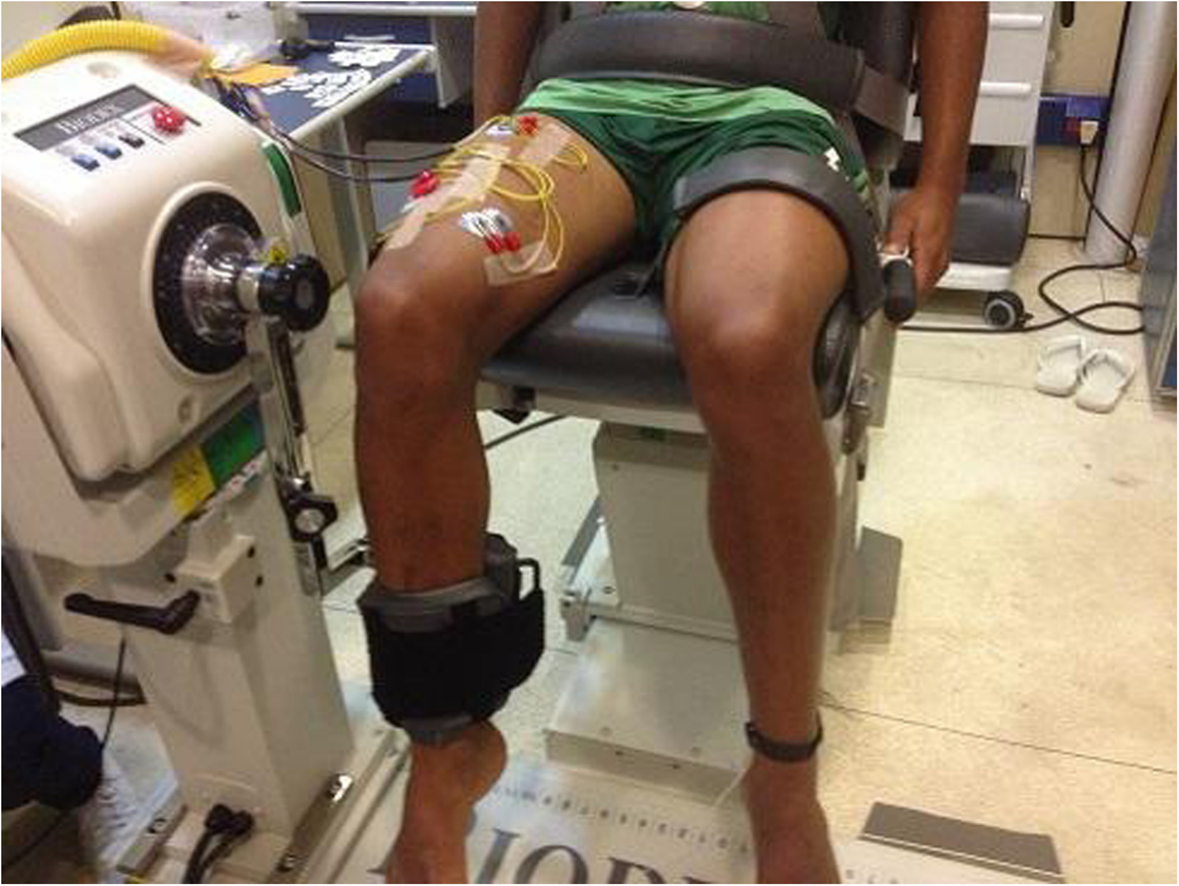
Experimental setup employed to record the EMG and the torque.

Sample results from an analysis using the *quadriceps femoris* muscle have been included for illustrative purposes. Data from *vastus medialis* (VM), *vastus lateralis* (VL) and *rectus femoris* (RF) were acquired at a 2 kHz sampling frequency in a young adult male. Figure 7 shows the experimental setup used to obtain the bipolar EMG signals and the isometric knee torque.

**Figure 8:**
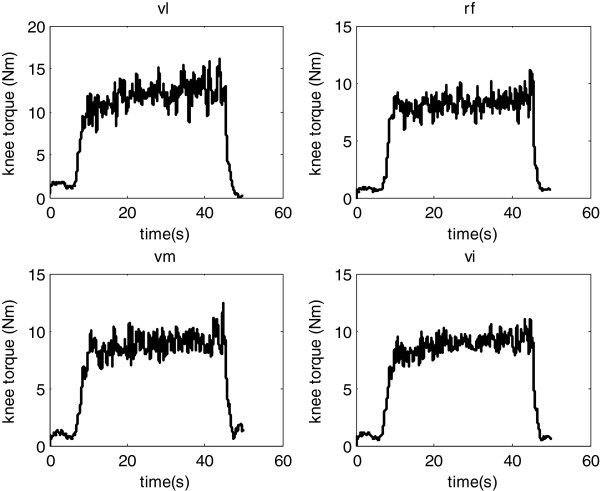
**Sample test data from quadriceps.** The torque contribution of each component is summed and compared to the total estimated knee torque.

**Figure 9:**
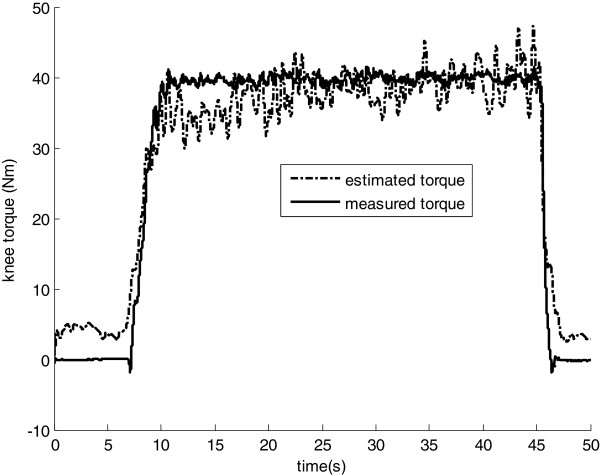
**Sample test data from quadriceps – torque.** Data are presented as a comparison between the EMG-driven-estimated and the dynamometer-measured torque.

The isometric contractions were performed with the knee flexed at 90° (see details in [21,22]). *Vastus intermedius* (VI) EMG activity was estimated as the average between *vastus medialis* (VM) and *lateralis* (VL), as in Figure 5. Figure 8 shows the individual contribution of each quadriceps component to the total knee extension torque in a step-following manoeuver: the subject tries to track a protocol mask that corresponds to a 30-second plateau of 20% of the MVC torque, preceded and followed by relaxing intervals. In Figure 9, the total knee torque estimated from the EMG signals is compared with the torque measured from the dynamometer.

The accuracy of individual muscle force estimation from EMG-driven models cannot be directly assessed. The joint torque error between the dynamometer measurements and the sum of the estimated partial muscle-force contributions is the best available index. For this particular experiment, the average RMS error between the estimated and the measured torque was 4 Nm, or approximately 10% of the plateau value.

The user should be aware that model estimation accuracy strongly depends on the choice of muscle parameters. The most influential is maximum muscle force, which varies widely among individuals. Some techniques can be applied to estimate this parameter individually (see details in [7,21,22,24]). The influence of ‘A’ parameter on the knee torque estimation accuracy was addressed in [21,22]. Optimisation of certain particular muscle model parameters, such as the maximum force, fibre length and tendon length, is a useful tool and easily decreases the torque error; however, it does not ensure that the ‘correct’ parameters values are always identified [15].

An important question regarding EMG-driven models is the estimation of a deep muscle from neighbouring superficial muscles, such as the *vastus intermedius* case shown here. Watanabe and Akima [25] studied the relationship among EMG activities from knee torque, *vastus intermedius* and other components of *quadriceps femoris*. Based on their data, we adjusted regression equations to estimate VI activity from the normalised torque; from the VM-, VL- and RF-measured EMGs; and as the average between VL and VM [22]. These regression equations were used to estimate the quadriceps components’ forces using the EMG-driven model. All estimation methods provided the same level of torque reconstruction accuracy, suggesting that the average between VL and VM is a reasonable estimation of VI EMG, at least for this particular application. Because the GUI can be applied to virtually any joint, the user must be aware of the hypotheses that are being taken into account to estimate the unmeasured EMG from synergistic muscles.

## Conclusions

This paper describes a Matlab application with a GUI that guides the user, step-by-step, to estimate the muscle forces from EMG signals. The forces are found by integrating a set of ODEs describing a Hill-type muscle model. The EMG envelope is considered the muscle excitation input. The model’s accuracy is assessed by comparing the estimated joint torque, which is calculated from the sum of the partial torque contributions provided by the muscles, with that measured using a dynamometer. The GUI also provides several types of outputs and analysis tools. Although lower limb muscle reference values are provided, the choice of muscle dynamics parameters depends on user discretionary. Force estimation accuracy is greatly sensitive on muscle parameter values and EMGD-FE can be useful for tuning these parameters.

A new version of the GUI is being developed to broaden the possible types of EMG-driven analysis, which are currently restricted to isometric tests. Non-isometric analysis requires describing the musculotendon length and the moment arms as a function of the joint angles. In addition, the Hill-type muscle model becomes especially sensitive to certain parameters, such as the tendon stiffness and the slack length, requiring previous calibrations based on optimisation methods.

## Availability and requirements

**Project name:** EMG Driven Force Estimator (EMGD-FE)

**Project home page:** to be defined

**Operating system(s):** Windows

**Programming language:** Matlab

**Other requirements:** Matlab Signal Processing Toolbox

**Any restrictions to use by non-academics:** no restrictions

## Endnotes

^a^The EMGD-FE v.1.0 package, including synchronized EMG and torque samples for *quadriceps femoris* and *triceps surae* and the User Manual can be freely downloaded from http://www.peb.ufrj.br/docentes/Luciano/EMG-FE.htm. Sample data for other muscles can be made available on a collaborative basis.

^b^The Matlab routine for filtering main hums noise has been developed by the Laboratorio di Ingegneria del Sistema Neuromuscolare e della Riabilitazione Motoria, Politecnico di Torino, Italy and is used with permission.

## Abbreviations

EMG: Electromyography; RF: *rectus femoris*; VM: *vastus medialis*; VL: *vastus lateralis*; VI: *vastus intermedius*; GUI: Graphical user interface; ODE: Ordinary differential equation; XML: Extensible Markup Language; MVC: Maximum voluntary contraction.

## Competing interests

The authors declare that they have no competing interests.

## Author’s informations

LLM is B.Sc., M.Sc. and Ph.D. in Mechanical Engineering. LFO is B.Sc. in Physiotherapy and Physical Education, M. Sc. and Ph.D. in Biomedical Engineering. Both are Associate Professors at the Biomedical Engineering Program of the Federal University of Rio de Janeiro. KKM is a mechanical engineer with background in computer programming and currently works with PETROBRAS Petroleum Company.

## Authors’ contributions

LLM developed the current formulation of the muscle model and is the Project coordinator. LFO collected the testing EMG samples and participated in the GUI procedure of analysis definitions. KKM implemented most of the Matlab code. All authors read and approved the final manuscript.

## References

[B1] Pandy MG Computer modeling and simulation of human movement Annual Rev Biomed Eng 2001 3 245 273 10.1146/annurev.bioeng.3.1.245 11447064

[B2] Ackermann M van den Bogert AJ Optimality principles for model-based prediction of human gait J Biomech 2010 43 1055 1060 10.1016/j.jbiomech.2009.12.012 20074736 PMC2849893

[B3] Nikooyan AA Veeger HEJ Westerhoff P Bolsterlee B Graichen F Bergmann G van der Helm FCT An EMG-driven musculoskeletal model of the shoulder Hum Mov Sci 2012 31 429 447 10.1016/j.humov.2011.08.006 22244106

[B4] Kumar D Rudolph KS Manal KT EMG-driven modeling approach to muscle force and joint load estimations: Case study in knee osteoarthritis J Orthopaedic Res 2012 30 377 383 10.1002/jor.21544 PMC3250571 21901754

[B5] Shao Q Macleod TD Manal KT Buchanan TS Estimation of ligament loading and anterior tibial translation in healthy and ACL-deficient knees during gait and the influence of increasing tibial slope using EMG-driven approach Ann Biomed Eng 2011 39 110 121 10.1007/s10439-010-0131-2 20683675 PMC3010217

[B6] Menegaldo LL Oliveira LF The influence of modeling hypothesis and experimental methodologies in the accuracy of muscle force estimation using EMG-driven models Multibody Sys Dyn 2012 28 21 36 10.1007/s11044-011-9273-8

[B7] Menegaldo LL Oliveira LF Effect of muscle model parameter scaling for isometric plantar flexion torque prediction J Biomech 2009 42 2597 2601 10.1016/j.jbiomech.2009.06.043 19665714

[B8] Shao Q Bassett DN Manal KT Buchanan TS An EMG-driven model to estimate muscle forces and joint moments in stroke patients Comp Biol Med 2009 29 1083 1088 10.1016/j.compbiomed.2009.09.002 PMC2784179 19818436

[B9] Lloyd DG Besier TF An EMG-driven musculoskeletal model to estimate muscle forces and knee joint moments in vivo J Biomech 2003 36 765 776 10.1016/S0021-9290(03)00010-1 12742444

[B10] Li L Tong KY Hu XL Hung LK Koo TKK Incorporating ultrasound-measured musculotendon parameters to subject-specific EMG-driven model to simulate voluntary elbow flexion for persons after stroke Clinic Biomech 2009 24 101 109 10.1016/j.clinbiomech.2008.08.008 19012998

[B11] Amarantini D Rao G Berton E A two-step EMG-and-optimization process to estimate muscle force during dynamic movement J Biomech 2010 43 1827 1830 10.1016/j.jbiomech.2010.02.025 20206935

[B12] Gerus P Rao G Berton E Subject-specific tendon-aponeurosis definition in hill-type model predicts higher muscle forces in dynamic tasks PLoS ONE 2012 7 8 e44406 10.1371/journal.pone.0044406 22952973 PMC3430662

[B13] Menegaldo LL Fleury AT Weber HI A ‘cheap’ optimal control approach to estimate muscle forces in musculoskeletal systems J Biomech 2006 39 1787 1795 10.1016/j.jbiomech.2005.05.029 16033695

[B14] Farina D Merletti R Enoka RM The extraction of neural strategies from the surface EMG J App Physiol 2004 96 1486 1495 10.1152/japplphysiol.01070.2003 15016793

[B15] Menegaldo LL Quadriceps Dynamic Model Tuning from Isokinetic Knee Torque using Optimization: a Numerical Simulation Study [abstract] Proceedings of Congress on Numerical Methods in Engineering 2013 Barcelona, Spain: Spanish Society of Numerical Methods in Engineering a136 Bilbao, Spain

[B16] Zajac FE Muscle and tendon: properties, models, scaling and application to biomechanics and motor control CRC Critic Rev Biomed Eng 1989 17 359 411 2676342

[B17] Delp SL Anderson FC Arnold AS Loan P Habib A John C Guendelman E Thelen DG OpenSim: Open-source software to create and analyze dynamic simulations of movement IEEE Trans Biomed Eng 2007 54 1940 1950 18018689 10.1109/TBME.2007.901024

[B18] Menegaldo LL Fleury AT Weber HI Moment arms and musculotendon lengths estimation for a three-dimensional lower-limb model J Biomech 2004 37 1447 1453 10.1016/j.jbiomech.2003.12.017 15275854

[B19] Manal KT Buchanan TS One-parameter neural activation to muscle activation model: estimating isometric joint moments from electromyograms J Biomech 2003 36 1197 1202 10.1016/S0021-9290(03)00152-0 12831746

[B20] Woods JJ Bigland-Ritchie B An anatomical/functional argument for the existence of both linear and nonlinear surface EMG/force relationships in human muscles Am J Phys Med 1983 62 287 299 6650674

[B21] Menegaldo LL Oliveira LF An EMG-driven model to evaluate quadriceps strengthening after an isokinetic training Procedia IUTAM 2011 2 131 141

[B22] Menegaldo LL Oliveira LF Estimation of vastus intermedius activity: the impact on torque prediction accuracy in a EMG-driven model of the knee [abstract] Proceedings of ISB 2011 Brussels, Belgium: International Society of Biomechanics

[B23] Vieira TM Matta TT Oliveira LF The varying properties of the EMG signal during isometric maximum voluntary contraction Mot Control 2007 11 70 71

[B24] Oliveira LF Menegaldo LL Individual-specific muscle maximum force estimation using ultrasound for ankle joint torque prediction using an EMG-driven Hill-type model J Biomech 2010 43 2816 2821 10.1016/j.jbiomech.2010.05.035 20541763

[B25] Watanabe K Akima H Normalized EMG to normalized torque relationship of vastus intermedius muscle during isometric knee extension Europ J App Physiol 2009 106 665 673 10.1007/s00421-009-1064-z 19404670

